# Acknowledgement to Reviewers of *Insects* in 2018

**DOI:** 10.3390/insects10010024

**Published:** 2019-01-09

**Authors:** Insects Editorial Office

**Affiliations:** MDPI, St. Alban-Anlage 66, 4052 Basel, Switzerland

Rigorous peer-review is the corner-stone of high-quality academic publishing. The editorial team greatly appreciates the reviewers who contributed their knowledge and expertise to the journal’s editorial process over the past 12 months. In 2018, a total of 196 papers were published in the journal, with a median time to first decision of 21 days and a median time to publication of 48 days. The editors would like to express their sincere gratitude to the following reviewers for their cooperation and dedication in 2018:
Abro, G. H.Luckhart, ShirleyAdang, MichaelLundin, OlaAddesso, KarlaMa, GuojiaAdelman, ZacharyMacias, Vanessa M.Ahn, JeongjoonMacLean, Heidi J.Akhtar, YasminMagura, TiborAlburaki, MohamedMainali, BishwoAlford, LucyMaistrello, LaraAllen, Margaret L.Malacrinò, AntoninoAlout, HaouesMalcolm, StephenAmiri, EsmaeilMandrioli, MauroAnderbrant, OlleManfredini, FabioAndersen, Jeremy C.Manino, AuloAnton, SylviaMankin, Richard W.Appel, Arthur G.Mann, Rajinder S.Arthurs, StevenMantilla-Contreras, JasminAthanassiou, ChristosMantzoukas, SpiridonAugustinos, AntoniosMarchant, RichardAvtzis, Dimitrios N.Marini, MarioAyayee, PaulMarshall, Katie E.Baker, MelissaMartel, VéroniqueBallardini, MarcoMartin, ArnaudBalzan, StefaniaMartin, StephenBarber-James, Helen M.Martínez, Luis CarlosBarman, ApurbaMartins, FabricioBattisti, AndreasMartín-Vega, DanielBaxter, SimonMassimino Cocuzza, GiuseppeBeaurepaire, AlexisMattei, CesarBeebe, NigelMaynard, DanielBenecke, MarkMazzoni, EmanueleBenedict, Mark Q.McCravy, Kenneth W.Bennett, Meghan M.McDonald, AllisonBerenbaum, May R.McEvoy, PeterBernardo, UmbertoMcFarland, KentBeyenbach, Klause W.McNeil, Jeremy N.Bierzychudek, PauletteMcNeill, CorraineBignell, DavidMeiklejohn, Kelly A.Bjørge, JulieMerchant, MichaelBlack, William C.Meuti, MeganBlacquière, TjeerdMichalik, AnnaBlair, CarolMilbrath, Meghan O.GradyBocak, LadislavMiles, Carol I.Bohman, BjörnMillán, AndrésBonali, Fabio LucaMiller, WolfgangBonilla-Rosso, GermánMilotic, TanjaBorges, Paulo A. V.Mitchell, Forrest L.Bose, JoyMlocicki, DanielBosso, LucianoMoar, William J.Brackney, DougMody, KarstenBradshaw, TerenceMogilicherla, KanakachariBrambila, JulietaMogren, ChristinaBrankatschk, MarkoMooney, Emily H.Breeuwer, Johannes A. J.Morales-Ramos, Juan A.Brent, ColinMorandin, ClaireBritch, S. C.Moreno, AránzazuBroughton, SonyaMori, BoydBrownbridge, MichaelMoriyama, MinoruBrückner, AdrianMorrison, William R.Brundage, AdrienneMorse, Douglass H.Brunet, Bryan M. T.Mound, Laurence A.Brutscher, Laura M.Moura, Luciano De AzevedoBryant, William B.Mullins, Aaron J.Buchanan, AmandaMurchie, Archie K.Buitenhuis, RoseMurillo, RosaBurgio, GiovanniMutinelli, FrancoBurkett-Cadena, NathanNagaoka, SumiharuBurrows, AndreaNarouei-Khandan, Hossein A.Calkins, TravisNavarro, ShlomoCampbell, EwanNedvěd, OldřichCampolo, OrlandoNeumann, PeterCampos-Herrera, RaquelNeupane, AvisheshCarrillo, DanielNielsen, AnneCastrejón-Gómez, Víctor RogelioNieslen, AnneChakrabarti Basu, PriyadarshiniNiño, ElinaChang, Chiou LingNishiwaki, HisashiChapman, NadineNogami, MakotoChapman, Nadine C.Noriyuki, SuzukiChappell, Thomas M.Nottingham, LouisChaves, LuisNugnes, FrancescoCheng, Sen-SungNumata, HideharuChevalier, MathieuNuss, Andrew B.Choe, Dong-HwanOi, David H.Chouvenc, ThomasOi, FaithChristiaens, OlivierOkada, KensukeChung, HenryO'Neal, ScottCiota, AlexanderOppenheim, SaraCivan, PeterOrtiz-Urquiza, AlmudenaClaborn, DavidOsada, YoheiConti, BarbaraOsbrink, Weste L.Cook, StevenOstiguy, NancyCopren, Kirsten A.Otuka, AkiraCorby-Harris, Vanessa LouiseOvergaard, JohannesCornel, AnthonyOzaki, KatsuhisaCortet, JérômePaploski, IgorCosme, Luciano V.Pappas, Maria L.Costa-Da-Silva, André LuisPark, Hyun-WooCostello, Michael J.Park, Il-KwonCridge, AndrewPark, Kye ChungCrozatier-Borde, MichelePark, Soo JeanCuthbertson, Andrew G. S.Patrick, MarjorieCzaczkes, Tomer JosephPavela, RomanCzarnoleski, MarcinPearson, GwenDai, Shu-MeiPerera, OmaththageDainat, BenjaminPeterson, Brittany F.Daniels, JaretPettersson, LarsDarbro, JonathanPfeiffer, DouglasDavis, Andrew K.Picimbon, Jean-FrançoisDE MEYER, MarcPickett, Charles H.De Souza, Danival JoséPirk, ChristianDeans, CarriePleasants, John MDelsinne, ThibautPondeville, EmilieDenux, OlivierPope, Karen L.Di Giulio, AndreaPopham, Holly J.Di Pasquale, GarancePortilla, MaribelDias, June FerrazPoudel, BarunDietrich, Chris H.Prasad, AbhishekDillman, AdlerQuerner, PascalDillman, Adler R.Rand, Tatyana A.Dindo, Maria LuisaRangel, JulianaDolezal, Adam G.Ranger, ChristopherDolezal, Adam G.Ravenscraft, AlisonDoorenweerd, CamielRazze, JanineDoremus, MatthewReddy, GadiDorin, AlanRehman, Junaid U.Doube, BernardRendon, DalilaDowling, AndreaRenkema, JustinDroege, SamRenkema, Justin M.Duan, JianRibeiro, JoséDupuis, JulianRicupero, MicheleDuso, CarloRiddick, EricDyer, AdrianRiehle, MichaelDyson, Paul J.Rijal, JhalendraEady, PaulRiley, David G.Eliopoulos, Panagiotis A.Rinkevich, FrankElkinton, JoeRobinson, Gene E.Endersby, Nancy MargaretRoditakis, EmmanouilEpsky, Nancy D.Rodriguero, MarcelaErram, DineshRodríguez-Rojas, AlexandroFahrbach, SusanRoggero, AngelaFeldhaar, Heike FeldhaarRomero, AlvaroFellowes, MarkRondoni, GabrieleFleming, DanielRoubik, David W.Flenniken, MichelleRoubos, Craig RForister, MattRouhier, Matthew F.Fornoff, FelixRoy, SouravFoster, WoodbridgeRuberson, John R.Foy, BrianRuiu, LucaFrank, Erik T.Rust, MichaelFrank, KevinRyabov, EugeneFreund, FriedemannRybska, ElizaFritz, Megan L.Sagili, RameshFunderburk, JoeSaigusa, KiyoshiG S Cuthbertson, AndrewSalis, LuciaGarcia, Flávio Roberto MelloSalom, ScottGendrin, MathildeSaloña-Bordas, Marta I.Gezon, ZakSan Jose, MichaelGhosh, SubirSánchez-Bayo, FranciscoGibbs, JasonSans, AlbertGile, GillianSantoiemma, GiacomoGill, Harsimran K.Sato, YukitaGiovenazzo, PierreSavvides, AndreasGisder, SebastianSbordoni, ValerioGiunti, GiuliaScharf, MichaelGloag, RosScheffrahn, Rudolf H.Goff, Gaëlle LeSchetelig, Marc F.Golick, DougScholtens, Brian G.Gomez-Marco, FrancescSeal, Dakshina R.Gondhalekar, Ameya D.Setamou, MamoudouGonzález-Zamora, Jose ESeverns, Paul M.Gotoh, HirokiSeymoure, Brett M.Grabenweger, GiselherShapiro, ArthurGrace, KennethShaw, Dana K.Grassl, JuliaShearer, Peter W.Greenslade, PenelopeShelly, ToddGregg, PeterShi, Yi-MingGriffin, ChristineShockley, MarianneGrof-Tisza, PatrickShostak, AllenGross, AaronShrestha, ManiGroßhans, JörgSial, Ashfaq AhmadGruner, SusanSiderhurst, MatthewGruwell, MatthewSiegel, JoelGrzywacz, AndrzejSilk, PeterGuastella, DevidSim, SheinaGuerra, Patrick AnthonySimoes, ZilaGuerrero, AngelSkelley, Paul E.Guis, HélèneSkorokhod, OleksiiHaddi, KhalidSkovgård, HenrikHaelewaters, DannySlipinski, AdamHalbert, SusanSmart, MatthewHall, DavidSmith, Hugh A.Hallem, Elissa A.Smith, Sarah M.Hamby, KellySmodiš Škerl, MajaHanly, Joseph J.Snow, JanathanHansen, AllisonSolensky, MichelleHarbison, JustinSparks, MichaelHart, AdamSpencer, Joseph L.Hassan, ErrolSpiteller, DieterHatt, SéverinSponsler, DouglasHealy, KristenSrinivasan, RamasamyHeerman, MatthewStark, John D.Hemerik, LiaSterzyńska, MariaHendriksma, HarmenStevens, Michael T.Heterick, Brian E.Stockan, JenniHill, GeenaStockton, DaraHill, MattStorer, Nicholas P.Hillyer, Julián F.Stout, MichaelHoffmann, ChristophStrand, Michael R.Hoffmann, KlausStrong, Donald R.Hogsette, JeromeStudebaker, Glenn E.Holden, Matthew H.Suh, EunhoHope, SandraSuiter, DanHorgan, Finbarr G.Surapathrudu, KanakalaHottel, BenjaminSusset, Eline C.Howlett, BradSweeney, JonHribar, LawrenceSwengel, Ann B.Hu, YiSwitzer, CallinHuang, XinSyed, ZainulabeuddinHuang, Yan-JangSzumny, Antoni JacekHuang, ZacharyTabata, JunHutchison, WilliamTai, HelenIbrahim, Sulaiman SadiTarasco, EustachioIngram, ErinTarasov, SergeiIsman, Murray B.Tatsuta, HarukiJacobsen, Stine KramerTaylor, DavidJaeger, HenryTetreau, GuillaumeJaenike, JohnThorn, SimonJaffe, BenjaminThrone, JamesJohnson, Kelly S.Todd, Jacqui H.Johnson, ReedTojo, KojiJohnston, J. SpencerTonkyn, DaveJones, CarolTosi, SimoneJones, DavidToussaint, Emmanuel F.A.Jones, David T.Traver, BrennaJones, JuliaTrdan, StanislavJoseph, ShimatTrematerra, PasqualeJoshi, Neelendra K.Trotter, TalbotJouquet, PascalTschinkel, Walter R.Judd, TimothyTucker, ErikaJung, ChuleuiTurell, Michael J.Jürgens, AndreasTzin, VeredKageyama, DaisukeUnbehend, MelanieKainoh, YooichiUrban, JulieKalsi, MeghaValles, StevenKang, SeokyoungVan Belleghem, StevenKaramaouna, FilitsaVan Der Kooi, CasperKarban, RichardVander Meer, RobertKawasaki, HidekiVankosky, Meghan A.Keena, MelodyVarloud, MarieKehrli, PatrikVelez, AnaKhadempour, LilyVerheggen, FrançoisKianifariz, MahnazVerhulst, NielsKim, DohyeongVerneau, FabioKirker, GrantVillalobos, Ethel M.Kiuchi, TakashiVillaverde, Juan JoséKleier, Daniel A.Vuts, JozsefKlingen, IngeborgWada-Katsumata, AyakoKnight, AlanWagler, RonaldKnisley, C. BarryWagner, SvenKohler, SteveWalgenbach, JamesKonagaya, TatsuroWalker, GregoryKooij, Pepijn W.Waller, DeborahKoou, Sin-YingWalton, William E.Koyama, TakashiWatari, YasuhikoKozlov, MikhailWebb, CameronKrischik, Vera A.Weeks, FaithKrishnan, NatrajWells, CarrieKristensen, Torsten NygårdWells, JeffreyKumar, PradeepWeston, PaulKumar, VivekWetterer, James K.Kuo, Ping-ChungWhitehorn, PenelopeKupper, MariaWiegmann, Brian M.Kvamme, TorsteinWiesner, RudolfKwong, Waldan K.Wiggins, GregLahiri, SriyankaWilhelm, GerthaLahondere, ChloeWilson, HoustonLamp, BenjaminWilson, Joseph S.Lampert, NiclasWilson, MichaelLander, TonyaWinder, LintonLapointe, StephenWink, MichaelLavista Llanos, SofíaWise, JohnLawler, SharonWone, BernardLazzari, ClaudioWoyke, JerzyLeather, SimonWright, DenisLecocq, AntoineWright, MarkLécureuil, CharlotteXu, GuangLee, Jana C.Xue, Rui-DeLee, Joon-hakYagound, BorisLees, Rosemary SusanYamanaka, NaokiLehmann, PhilippYanagawa, AyaLester, P. J.Yaninek, SteveLiao, Ling-HsiuYao, JianxiuLinnakoski, RiikkaYashiro, ToshihisaLobo, NeilYee, Wee L.Loni, AugustoYoshioka, AkiraLooney, ChrisZhang, Qing-HeLopez-Ferber, MiguelZografou, KonstantinaLópez-López, AlejandroZolnerowich, Gregory

